# Mixture of Peanut Skin Extract, Geniposide, and Isoquercitrin Improves the Hepatic Lipid Accumulation of Mice via Modification of Gut Microbiota Homeostasis and the TLR4 and AMPK Signaling Pathways

**DOI:** 10.3390/ijms242316684

**Published:** 2023-11-24

**Authors:** Meijuan Yi, Opeyemi B. Fasina, Yajing Li, Lan Xiang, Jianhua Qi

**Affiliations:** 1 College of Pharmaceutical Science, Zhejiang University, Hangzhou 310058, China; 22119153@zju.edu.cn (M.Y.); fasinaopeyemi@zju.edu.cn (O.B.F.); 12019045@zju.edu.cn (Y.L.); 2 Jinhua Institute of Zhejiang University, Jinhua 321299, China

**Keywords:** metabolic-dysfunction-associated steatotic liver disease, TLR4/NF-κB, AMPK, inflammation, gut microbiota

## Abstract

Metabolic-dysfunction-associated steatotic liver disease (MASLD, formerly known as NAFLD) is a global chronic liver disease, and no licensed drugs are currently available for its treatment. The incidence of MASLD is increasing, which could lead to a huge clinical and economic burden. As a multifactorial disease, MASLD involves a complex set of metabolic changes, and many monotherapies for it are not clinically effective. Therefore, combination therapies using multiple drugs are emerging, with the advantages of improving drug efficacy and reducing side effects. Peanut skin extract (PSE), geniposide (GEN), and isoquercitrin (IQ) are three natural antiaging components or compounds. In this study, the preventive effects of individual PSE, GEN, and IQ in comparison with the effects of their mixture (MPGI) were examined in a mouse model of high-fat-feed-induced MASLD. The results showed that MPGI could significantly reduce the body and liver weights of mice and improve hepatic steatosis and liver function indicators. Further mechanistic studies showed that PSE, GEN, and IQ worked together by reducing inflammation, modulating the intestinal flora, and regulating the TLR4/NF-κB, AMPK/ACC/CPT1, and AMPK/UKL1/LC3B signaling pathways. It is a promising therapeutic method for preventing MASLD.

## 1. Introduction

Non-alcoholic fatty liver disease (NAFLD) is a global chronic liver disease [1], in which at least 5% of an individual’s hepatocyte infiltrates are diagnosed with steatosis via imaging or liver biopsy [2]. Currently, content experts are advocating forthe nomenclature of NAFLD to change to metabolic-dysfunction-associated steatotic liver disease (MASLD) in order to describe the aetiology of the disease more accurately [3]. Its main pathogenesis is the entry of excess energy into the liver, leading to a net accumulation of energy in the liver in the form of triglycerides (TG) [2,4]. MASLD ranges from a simple steatotic liver to metabolic-dysfunction-associated steatohepatitis (MASH), cirrhosis, and eventually hepatocellular carcinoma (HCC) as the disease progresses [3,4]. A report showed that 25% of the global population is currently considered to have MASLD [5], and recent data indicated that the burden of MASLD has increased dramatically over the past two decades in Asia [6]. However, no licensed drugs are currently available for treating MASH [7]. Although diet and exercise have been shown to be effective in treating MASLD, the long-term sustainability of these interventions is poor [8]. The incidence of MASLD could continue to increase, which could lead to a huge clinical and economic burden [9]. Therefore, effective drugs for MASLD are urgently needed.

MASLD is a disease with multiple causative factors that result in a complex array of metabolic changes [2,10]. Extensive clinical evidence has indicated that MASLD is not only linked to an increased risk of liver-related morbidity and mortality but also to the development of other remarkable extra-hepatic disorders [10], which include cardiovascular disease, obesity, insulin resistance, type 2 diabetes mellitus, and chronic kidney disease [10]. Therefore, many monotherapies for MASLD are not clinically effective, with no more than 40% of patients benefiting from monotherapy [11]. As a result, combination therapies using multiple drugs are emerging, with the advantages of improving drug efficacy and reducing side effects [12].

The gut–liver axis, which communicates bi-directionally through the biliary tract, the portal vein, and systemic circulation, plays an important role in MASLD [13]. Meanwhile, alterations in the composition of the gut microbiome and microbe-derived metabolites are associated with the progression of MASLD [14]. Moreover, the increase in intestinal permeability caused by the disruption of the intestinal barrier leads to excessive influx of lipopolysaccharide (LPS) and some other microbial metabolites into the blood and liver, which also leads to MASLD [15,16].

Natural products have been the main sources of new drugs [17]. Peanut skin extract (PSE), geniposide (GEN), and isoquercitrin (IQ) are three parts that have novel anti-aging effects and a clear chemical composition, structure, and mechanism of action, which have been identified in our previous studies. Peanut skin is a thin, red skin found on the outside of peanuts, and it is utilized for the treatment of hemophilia, primary and secondary thrombocytopenic purpura, and hepatic hemorrhage in traditional Chinese medicine. PSE is extracted from peanut skin and mainly includes oligosaccharides and flavonoids. It has shown anti-obesity and anti-diabetic effects on obese and diabetic mice in previous studies [18,19]. GEN is a cyclohexene glucoside and the main active ingredient in Gardenia, and it has many activities, including neuroprotective, antidiabetic, hepato-protective, anti-inflammatory, cardioprotective, antioxidant, and antitumoral effects [20]. IQ is a flavonoid substance derived from *Apocynum venetum* L. that exerts anti-obesity effects in obese mice by targeting C-1-tetrahydrofolate synthase, carbonyl reductase, and glutathione S-transferase P and modifying the AMPK/SREBP-1c/FAS/CD36 signaling pathway [21]. Since the pathogenesis of MASLD and aging have common characteristics, such as oxidative stress and inflammation [2,22], we consider that anti-aging compounds and fraction may have an effect on MASLD. To demonstrate this hypothesis, MPGI was utilized to treat MASLD in this study. Here, we report that MPGI can effectively improve the hepatic steatosis and liver functions of high-fat diet (HFD)-fed mice by regulating multiple pathways, including gut microbiota homeostasis and the LPS/TLR4/NF-κB, AMPK/ACC/CPT1A, and AMPK/ULK1/LC3B signaling pathways.

## 2. Results

### 2.1. MPGI Extends the Replicative Lifespan of K6001 Yeast and Inhibits the Eto-Induced Senescence of PC12 Cells

**Figure 1:**
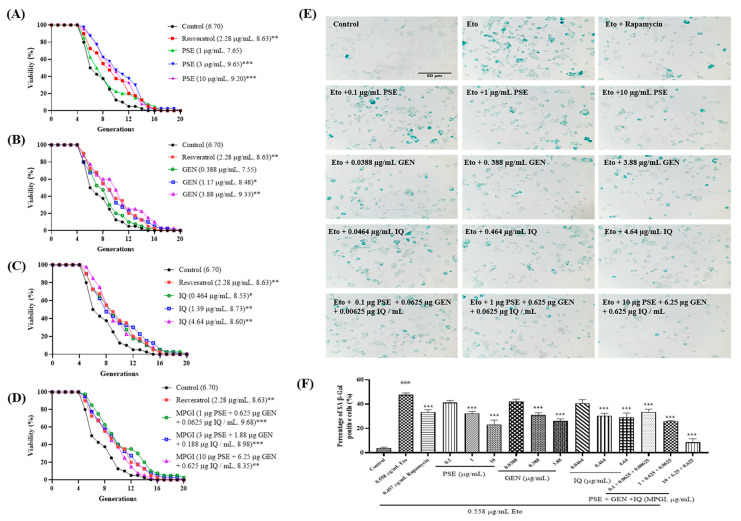
MPGI extends the replicative lifespan of K6001 yeast and inhibits Eto-induced senescence in PC12 cells. The results of a replicative lifespan assay of PSE (**A**), GEN (**B**), IQ (**C**), and MPGI (**D**). *, **, and *** indicate significant differences at *p* < 0.05, *p* < 0.01, and *p* < 0.001 compared with the control group in (**A**) to (**D**). The anti-aging effect of PSE, GEN, IQ, and MPGI on senescence in PC12 cells induced by Eto (**E**) and their digital results (**F**). The data are presented as mean ± SEM. ^###^ indicates significant difference at *p* < 0.001 compared with the control group in (**E**). *** represents significant difference at *p* < 0.001 compared with the Eto group in (**E**).

Aging and MASLD involve many common disease factors and mechanisms, such as inflammation, autophagy, and metabolic syndrome [2,22]. Therefore, the antiaging activities of PSE, GEN, and IQ alone and their combination in yeast and PC12 cells were determined. The results of the replicative lifespan assay of K6001 yeast are shown in Figure 1A–D. PSE, GEN, and IQ significantly extended the replicative lifespan of K6001 yeast at concentrations of 3 and 10 μg/mL (Figure 1A; *p* < 0.001 and *p* < 0.001 respectively), 1.17 and 3.88 μg/mL (Figure 1B; *p* < 0.05 and *p* < 0.01, respectively), and 1.39 and 4.64 μg/mL (Figure 1C; *p* < 0.01 and *p* < 0.01, respectively). In addition, MPGI significantly extended the replicative lifespan of K6001 yeast at concentrations of 1 μg/mL PSE + 0.625 μg/mL GEN + 0.0625 μg/mL IQ and 3 μg/mL PSE + 1.88 μg/mL GEN + 0.188 μg/mL IQ (Figure 1D; *p* < 0.001, *p* < 0.001, and *p* < 0.01, respectively). The average generation of a dose of the mixture with a low concentration was higher than that of the three components acting alone (Figure 1A–D). These results suggested that MPGI, PSE, GEN, and IQ have antiaging effects on yeast. Meanwhile, Etoposide (Eto) was used to induce senescence in PC12 cells, and the antiaging effects of PSE, GEN, IQ, and MPGI were examined using the SA β-Gal kit to determine whether they have the same antiaging effect on mammal cells. As expected, SA β-Gal-positive cells in the Eto group obviously increased (Figure 1E,F; *p* < 0.001), whereas those in the PSE, GEN, IQ, and MPGI groups significantly decreased (Figure 1E,F; *p* < 0.001 for PSE; *p* < 0.001 for GEN; *p* < 0.001 for IQ; *p* < 0.001 for MPGI). The percentages of senescence cells in the negative control and Eto groups were 3.82% and 47.68%, respectively. Meanwhile, the number of SA β-Gal-positive cells decreased in a dose-dependent manner when adding different concentrations of PSE, GEN, IQ, and MPGI (Figure 1E,F; *p* < 0.001, *p* < 0.001, *p* < 0.001, *p* < 0.001, *p* < 0.001, *p* < 0.001, *p* < 0.001, *p* < 0.001, and *p* < 0.001). These results indicated that PSE, GEN, IQ, and MPGI had the same antiaging effect on yeast and mammal cells, for which MPGI demonstrated the best effect.

### 2.2. MPGI Reduces Body Weight and Improves Hepatic Lipid Accumulation in MASLD Mice

**Figure 2:**
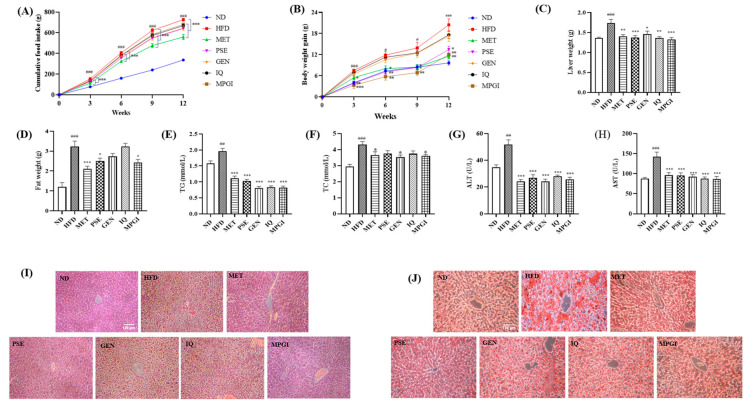
MPGI reduces body weight and improves hepatic lipid accumulation in mice with MASLD. Changes in the food intake (**A**), body weight gain (**B**), liver weight (**C**), and fat weight of the mice after treatment with MET, PSE, GEN, IQ, and MPGI (**D**). Changes in plasma TG (**E**), TC (**F**), ALT (**G**), and AST (**H**) of mice. The number of the mice in each group was seven. Microphotograph of liver tissue with H&E staining (**I**) and oil red O staining (**J**). The livers of three mice in each group were cut and stained. The data are presented as mean ± SEM. *, **, and *** indicate significant difference at *p* < 0.05, *p* < 0.01, and *p* < 0.001 compared with the HFD group; ^#^, ^##^, and ^###^ indicate significant difference at *p* < 0.05, *p* < 0.01, and *p* < 0.001 compared with the normal diet (ND) group.

Next, given the antiaging activities of PSE, GEN, and IQ and the many common disease factors and mechanisms involved in aging and MASLD, MPGI’s ability to improve hepatic steatosis in high-fat diet (HFD)-fed mice was evaluated. The food intake and body weight of the HFD group significantly increased compared with those of the normal control group (Figure 2A,B; *p* < 0.001 and *p* < 0.001). After 140 mg/kg metformin (MET), 80 mg/kg PSE, 50 mg/kg GEN, or 5 mg/kg IQ or MPGI was administered for 12 weeks, and the food intake of all the treatment groups obviously decreased at 3, 6, 9, and 12 weeks compared with that of the HFD group (Figure 2A; *p* < 0.001, *p* < 0.001, *p* < 0.001, and *p* < 0.001, respectively). The significant increase in body weight induced by HFD in the MET, PSE, and MPGI groups remarkably recovered to normal level (Figure 2B, *p* < 0.01, *p* < 0.05, *p* < 0.01). However, these effects on food intake and body weight were not observed in the GEN and IQ groups (Figure 2A,B). Except for the fat weight in the GEN and IQ groups, the fat and liver weights in all other treatment groups significantly decreased compared with those in the HFD group (Figure 2C: *p* < 0.01, *p* < 0.001, *p* < 0.05, *p* < 0.01, and *p* < 0.001, respectively; Figure 2D: *p* < 0.001, *p* < 0.05, and *p* < 0.05, respectively). Meanwhile, the blood biochemical indicators of mice were recorded. The plasma TG, TC, ALT, and AST of the HFD group significantly increased compared with those of the normal group, whereas those of the MET, GEN, and MPGI groups significantly decreased (Figure 2E–H; *p* < 0.001, *p* < 0.05, *p* < 0.001, and *p* < 0.001, respectively). Furthermore, H&E staining and oil red O staining were applied to detect any pathological changes in the mouse livers. In the HFD group, significant vacuolar degeneration and fat accumulation were observed. After treatment with MET, PSE, GEN, IQ, and MPGI, these pathological changes in the MET and MPGI groups demonstrated a unique improvement (Figure 2I,J). The results suggested that MPGI improved the hepatic steatosis of mice induced by HFD, and the effect of MPGI was equivalent to that of MET.

### 2.3. MPGI Alters the Composition of the Gut Microbiome in Mice with MASLD

**Figure 3:**
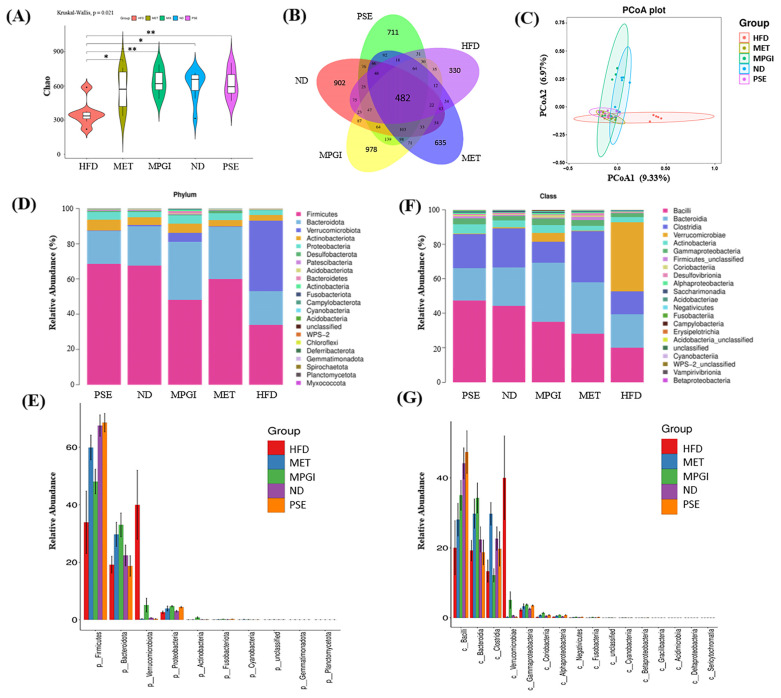
Effect of MPGI on the gut microbiota of mice with MASLD. Effect of MPGI on the gut microbiota of mice with MASLD at Chao of alpha diversity (**A**). Venn diagram of the number of ASVs common and unique to each group (**B**). Principal coordinate analysis of gut microbiota of mice with MASLD (**C**). Relative abundance of gut microflora and significantly changing gut microbiota at phylum (**D**,**E**) and class (**F**,**G**) levels. The number of samples in each group is seven. * and ** indicate significant difference at *p* < 0.05 and *p* < 0.01 compared with the normal control group or HFD group.

The gut–liver axis plays an important role in the development of MASLD [13]. Illumina high-throughput sequencing was conducted to read the 16S RNA sequences of the V4 region of fecal microflora samples of the ND, HFD, MET, PSE, and MPGI groups to understand whether MPGI affects the gut microbiota. The results of the α diversity analysis of mouse gut microbiota are shown in Figure 3A,B. The total number of species and the species richness of the gut microbiota in the HFD group were significantly reduced at Chao levels (Figure 3A; *p* < 0.05), whereas these parameters in the MET, PSE, and MPGI groups were restored to the same level as the ND group (Figure 3A; *p* < 0.05, *p* < 0.01, and *p* < 0.01, respectively). The Venn diagram illustrates the number of ASVs common and unique to each group. A total of 482 ASVs were common to the five groups, whereas 902, 330, 635, 711, and 978 ASVs were unique to the ND, HFD, MET, PSE, and MPGI groups, respectively (Figure 3B). The results of principal coordinate analysis (PCoA) are given in Figure 3C. The intestinal flora composition of the HFD group obviously changed, whereas that of the treatment groups recovered to a level close to that of the ND group. Amongst them, the intestinal flora composition of the MPGI group was the closest to the ND group. These results suggested that gut dysbiosis was reversed by MPGI, MET, and PSE. The relative abundance at the phylum and class levels was analyzed to explore the effect of MPGI on the relative abundance of microbes in the gut. The results of phylum analysis are displayed in Figure 3D,E. The HFD group showed a significant decrease in *Firmicutes*, *Fusobacteriota*, *Bacteroidetes*, *Proteobacteria*, *Cyanobacteria,* and *Unclassified* compared with the normal control (*p* < 0.05, *p* < 0.05, *p* < 0.05, *p* < 0.05, and *p* < 0.05, respectively). The relative abundance of *Verrucomicrobiota* in the HFD group was surprisingly high (over 40%), whereas the relative abundance of the other species in this group were less abundant than in other groups (*p* < 0.001). Meanwhile, the MET group exhibited a significant increase in *Firmicutes*, *Proteobacteria*, *Bacteroidetes*, *Cyanobacteria* and *Fusobacteriota* (*p* < 0.05, *p* < 0.05, *p* < 0.05, *p* < 0.05, and *p* < 0.05, respectively) and a significant reduction in *Unclassified* (*p* < 0.05) compared with the HFD group. After MPGI and PSE were administered, a marked increase in the relative abundance of *Firmicutes* and *Fusobacteriota* (*p* < 0.05 and *p* < 0.05, respectively), and a significant reduction in *Verrucomicrobia* (*p* < 0.001) were observed in the MPGI group compared with the HFD group. Besides *Bacteroidetes*, the same changes to these gut microbiotas were found in the PSE group. The results of class analysis are shown in Figure 3F,G. Compared with the normal control, the HFD group showed a significant increase in the relative abundance of *Verrucomicrobiae* (*p* < 0.001) and a significant decrease in that of *Clostridia, Bacilli*, *Bacteroidia*, *Negativicutes*, *Coriobacteriia*, *Gracilibacteria*, *Fusobacteriia,* and *Betaproteobacteria* (*p* < 0.05, *p* < 0.05, *p* < 0.05, and *p* < 0.05, respectively). Meanwhile, compared with the HFD group, the MET, PSE, and MPGI groups showed a significant reduction in the relative abundance of *Verrucomicrobiae* (*p* < 0.001) and a significant increase in that of *Clostridia*, *Bacilli*, *Negativicutes*, *Coriobacteriia*, *Gracilibacteria*, *Fusobacteriia,* and *Betaproteobacteria* (*p* < 0.05, *p* < 0.05, *p* < 0.05, *p* < 0.05, *p* < 0.05, *p* < 0.05, and *p* < 0.05, respectively). The increase in *Bacteroidia* was only observed in the MET and MPGI groups. These results indicated that MPGI significantly decreased the relative abundance of *Verrucomicrobiae* and increased that of *Bacteroidetes*, *Proteobacteria*, *Cyanobacteria,* and *Unclassified* at the phylum and class levels.

### 2.4. Function and Bacterial Phenotypic Prediction for the Gut Microbiota of Mice with MASLD

**Figure 4:**
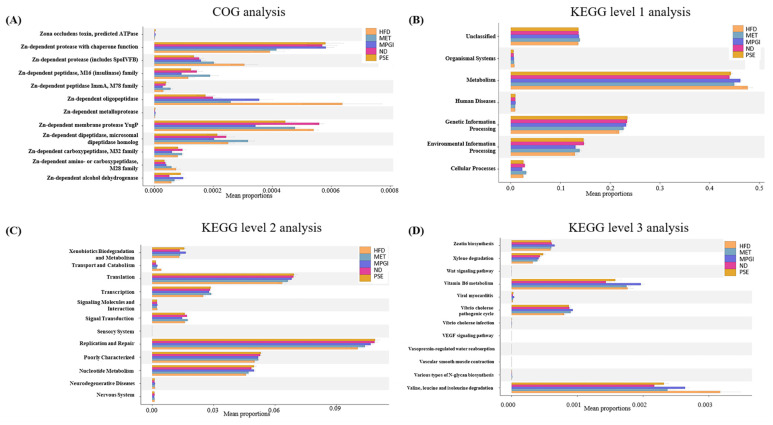
The function prediction of gut microbiota in mice with MASLD after treatment with MPGI. The results of COG analysis (**A**), KEGG analysis at level 1 (**B**), KEGG analysis at level 2 (**C**), and KEGG analysis at level 3 (**D**).

PICRUSts2 was used to conduct the function prediction of the gut microbiota. MPGI was found to mainly affect Zn-dependent enzymes to prevent MASLD in obese mice (Figure 4A), and it is involved in metabolic processes, genetic information processing, environmental information processing and cellular processes (Figure 4B). Furthermore, MPGI can be seen to mainly regulate xenobiotics biodegradation, transport and transcription, signal transduction, replication and repair, and poorly characterized nucleotide metabolism at level 2 of KEGG analysis (Figure 4C). In level 3 of KEGG analysis, a reduction in xylene degradation and *Vibrio cholera* pathogenic cycle and an increase in valine, leucine, and isoleucine degradation were observed in the HFD group compared with the normal control. After MPGI was administered, an increase in zeatin biosynthesis, xylene degradation, vitamin B metabolism, viral myocarditis, and the *V. cholera* pathogenic cycle and a reduction in valine, leucine, and isoleucine degradation were very apparent in the MPGI group (Figure 4D). In addition, pathway analysis was conducted, and the results are displayed in Supplementary Figure S1. MPGI was shown to inhibit urea cycle and stimulate the urate biosynthesis, UMP biosynthesis, UDP-N-acetylmuramoyl-pentapeptide, UDP-N-Acetyl-D-glucosamine biosynthesis I, and ubiquino-8,9 biosynthesis signaling pathways.

**Figure 5:**
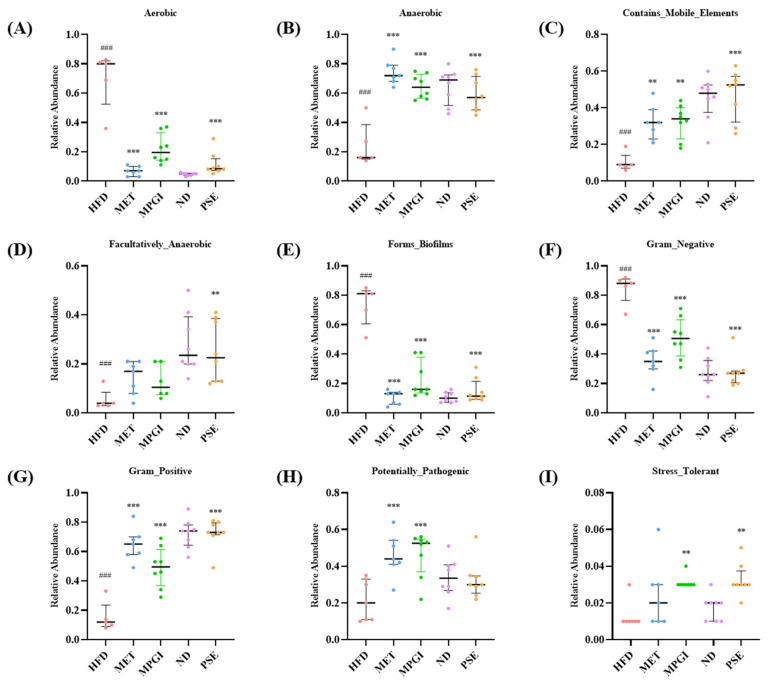
The bacterial phenotypic prediction of gut microbiota of mice with MASLD after treatment with MPGI. The changes in aerobic bacteria (**A**), anaerobic bacteria (**B**), contains_mobile_elements (**C**), facultatively_ anaerobic (**D**), bacteria that forms biofilms (**E**), Gram_negative bacteria (**F**), Gram_positive bacteria (**G**), potentially pathogenic bacteria (**H**) and stress_tolerant bacteria (**I**). The number of samples in each group is five or seven. ^###^ indicates significant difference at *p* < 0.001 compared with the ND group. ** and *** indicate significant difference at *p* < 0.01 and *p* < 0.001 compared with the HFD group.

Meanwhile, bacterial phenotypic prediction of the gut microbiota was performed. A reduction in bacteria that contain mobile elements and anaerobic, facultatively_anaerobic, and Gram_positive bacteria (Figure 5B, *p* < 0.001; Figure 5C, *p* < 0.001; Figure 5D, *p* < 0.001; Figure 5G, *p* < 0.001) and an increase in bacteria that forms biofilms and aerobic and Gram_negative bacteria (Figure 5A, *p* < 0.001; Figure 5E, *p* < 0.001; Figure 5F, *p* < 0.001) were observed in the HFD group compared with the normal control. Since the *Verrucomicrobiota* in the HFD group was considered to be beneficial microflora, the *Potentially_Pathogentic* bacteria in the MET and MPGI groups were higher than that of the HFD group (Figure 5H; *p* < 0.001 and *p* < 0.001, respectively). These parameters returned to normal levels in the MET, PSE, and MPGI groups (Figure 5A–I). These results confirmed that the microbiome–gut–liver axis is involved in the protective effect of MPGI for the liver.

### 2.5. MPGI Mitigates Inflammation of Mice with MASLD

**Figure 6:**
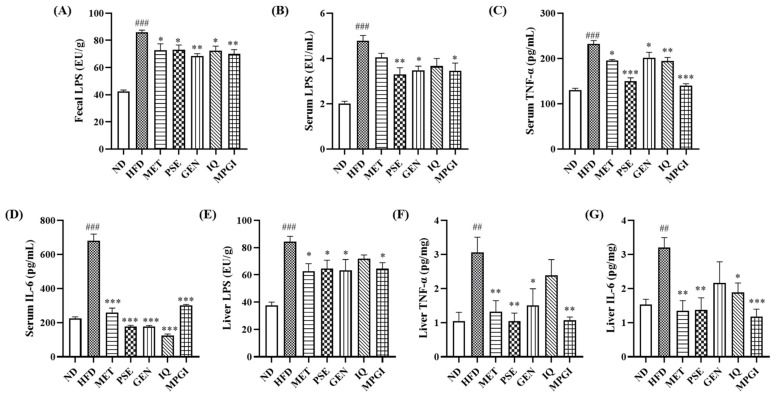
MPGI mitigates inflammation in MASLD mice. Changes in fecal LPS (**A**), serum LPS (**B**), serum TNF-α (**C**), serum IL-6 (**D**), LPS in liver (**E**), TNF-α in liver (**F**), and IL-6 in liver (**G**). The number of samples in each group is six. The data are presented as mean ± SEM. ^##^ and ^###^ indicate significant difference at *p* < 0.01 and *p* < 0.001 compared with the ND group; *, **, and *** indicate significant difference at *p* < 0.05, *p* < 0.01, and *p* < 0.001 compared with the HFD group.

Gut dysbiosis has been implicated in promoting increased LPS levels in the blood, which induces inflammation in marginal tissues and organs [15,16]. Therefore, the signaling pathway of inflammation was investigated. Fecal LPS and plasma LPS in the HFD group were significantly higher than those in the normal control (Figure 6A,B; *p* < 0.001 and *p* < 0.001, respectively). After MET, PSE, GEN, IQ, and MPGI were administered, fecal LPS in the MET, GEN, IQ, and MPGI groups and plasma LPS in the PSE, GEN, and MPGI groups were found to be lower than those of the HFD group (Figure 6A: *p* < 0.05, *p* < 0.05, *p* < 0.01, *p* < 0.05, and *p* < 0.01, respectively; Figure 6B: *p* < 0.01, *p* < 0.05, and *p* < 0.05, respectively). Furthermore, the proinflammatory cytokines in the plasma were measured, and the results are displayed in Figure 6C,D. Increases in plasma TNF-α and IL-6 in the HFD group and reductions in the treatment groups were also observed (Figure 6C: *p* < 0.001, *p* < 0.05, *p* < 0.001, *p* < 0.05, *p* < 0.01, and *p* < 0.001, respectively; Figure 6D: *p* < 0.001, *p* < 0.001, *p* < 0.001, *p* < 0.001, *p* < 0.001, and *p* < 0.001, respectively). These parameters were also examined in the liver, and the results are shown in Figure 6E–G. Compared with the normal control or HFD groups, the HFD group showed a significant increase in LPS (Figure 6E, *p* < 0.001), and the MET, PSE, and MPGI groups all showed reductions in LPS (Figure 6E; *p* < 0.05, *p* < 0.05, and *p* < 0.05, respectively). The proinflammatory cytokines, such as TNF-α and IL-6, were significantly increased in the livers of the HFD group but were decreased in all treatment groups (Figure 6F: *p* < 0.01, *p* < 0.01, *p* < 0.01, *p* < 0.05, and *p* < 0.01, respectively; Figure 6H: *p* < 0.01, *p* < 0.01, *p* < 0.01, *p* < 0.05, and *p* < 0.001, respectively). These results revealed that MPGI could mitigate inflammation in mice induced by HFD via attenuating endotoxin and proinflammatory cytokines in the plasma and liver.

### 2.6. MPGI Improves Inflammation by Regulating the Gut Microbiota and TLR4/NF-κB Signaling Pathway

**Figure 7:**
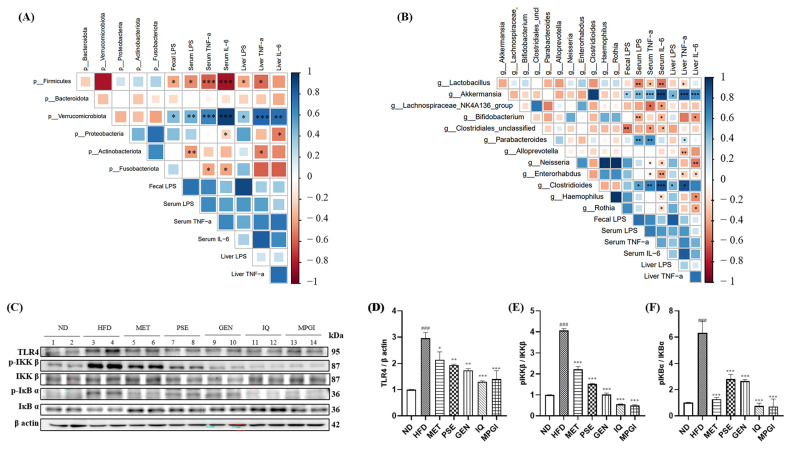
Correlation between gut microbiota and inflammation factors, and the effect of MPGI on the TLR4/NF-κB signaling pathway in the liver of mice with MASLD. Correlation between gut microbiota and inflammation factors at phylum level (**A**) and genus level (**B**). Western blot analysis of liver TLR4, pIKK β, IKK β, pIκB α, and IκB α (**C**). ND group, 1–2 wells; HFD group, 3–4 wells; MET group, 5–6 wells; PSE group, 7–8 wells; GEN group, 9–10 wells; IQ group, 11–12 wells; MPGI group, 13–14 wells. Each sample is a mixture of three independent mouse livers for each group. Digitalized results of the Western blot analysis (**D**–**F**). The data are presented as mean ± SEM. ^###^ indicates significant difference at *p* < 0.001 compared with the ND group; *, **, and *** indicate significant difference at *p* < 0.05, *p* < 0.01, and *p* < 0.001 compared with the HFD group.

Correlation analysis was performed at the phylum and genus levels by using a software to indicate whether a correlation exists between the abundance of gut microbiota and inflammatory factors. The *Firmicutes*, *Verrucomicrobiota*, *Proteobacteria*, *Actinobacteriota,* and *Fusobacteriota* at the phylum level were significantly correlated with LPS, TNF-α, and IL-6 in feces, serum, and liver (Figure 7A). *Firmicutes* exhibited a negative correlation with LPS in feces, serum, and liver (*p* < 0.05, *p* < 0.05, and *p* < 0.05, respectively) and with TNF-α and IL-6 in serum (*p* < 0.001 and *p* < 0.001, respectively). *Verrucomicrobiota* showed a positive correlation with LPS (*p* < 0.05, *p* < 0.01, and *p* < 0.05, respectively), TNF-α (*p* < 0.001 and *p* < 0.001, respectively), and IL-6 (*p* < 0.001 and *p* < 0.01, respectively) in feces, serum, and liver (*p* < 0.001 and *p* < 0.001, respectively). *Proteobacteria* was negatively correlated with IL-6 in serum and liver (*p* < 0.05 and *p* < 0.01, respectively), and *Actinobacteriota* was negatively correlated with LPS in serum (*p* < 0.01) and with TNF-α in liver (*p* < 0.05). *Fusobacteriota* had negative correlation with TNF-α and IL-6 in serum (*p* < 0.05 and *p* < 0.05, respectively) and liver (*p* < 0.05 and *p* < 0.05, respectively). Furthermore, *Lactobacillus*, *Lachnospiraceae*_NK4A136_group, *Bifidobacterium*, *Clostridiales*_*unclassified,* and *Clostridioides* were obviously associated with levels of inflammation at the genus level (Figure 7B). *Akkermansia* and *Clostridioids* demonstrated varying degrees of positive correlation with LPS, TNF-α and IL-6 in feces, serum, and liver. *Parabacteroides* showed positive correlation with LPS and TNF-α in serum and TNF-α in liver. Meanwhile, the microbiota that were negatively correlated with TNF-α and IL-6 in serum were *Lactobacillus*, *Lachnospiraceae*_NK4A136_group, *Clostridiales*_unclassified, *Neisseria,* and *Enterorhabdus,* and those negatively correlated with TNF-α in liver were *Lactobacillus*, *Bifidobacterium*, *Alloprevotella,* and *Enterorhabdus.* Furthermore, *Neisseria*, *Haemophilus,* and *Rothia* were negatively correlated with IL-6 in liver. These results suggest that the dysfunction in gut microflora and its metabolites is involved in inflammation in mice, and maintaining the hemostasis of gut microbiota may be an important measure in preventing MASLD.

In addition, how MPGI could reduce liver inflammation in MASLD mice was explored. Considering that an increase in LPS has been shown to activate the TLR4 signaling pathway, which increases the expression of proinflammatory markers, LPS may activate the TLR4/NF-κB signaling pathway in the liver of mice [23]. Thus, changes in this signaling pathway in the liver were determined. The protein levels of TLR4, phosphorylated IKK-β, and phosphorylated IκB-α in the HFD group significantly increased compared with those in the ND group (Figure 7C–F and Figure S2; *p* < 0.001, *p* < 0.001, and *p* < 0.001, respectively). On the contrary, these parameters in the MPGI group and other groups were decreased, and the effect of MPGI was found to be the most efficient (Figure 7D: *p* < 0.05, *p* < 0.01, *p* < 0.01, *p* < 0.001, and *p* < 0.001, respectively; Figure 7E: *p* < 0.001; *p* < 0.001, *p* < 0.001, *p* < 0.001, and *p* < 0.001, respectively; Figure 7F: *p* < 0.001; *p* < 0.001, *p* < 0.001, *p* < 0.001, and *p* < 0.001, respectively). The results suggest that MPGI improves liver inflammation via the regulation of LPS and the TLR4/NF-κB signaling pathway.

### 2.7. MPGI Regulates AMPK/ACC/CPT1 and Autophagy Signaling Pathways in the Liver of MASLD Mice

**Figure 8:**
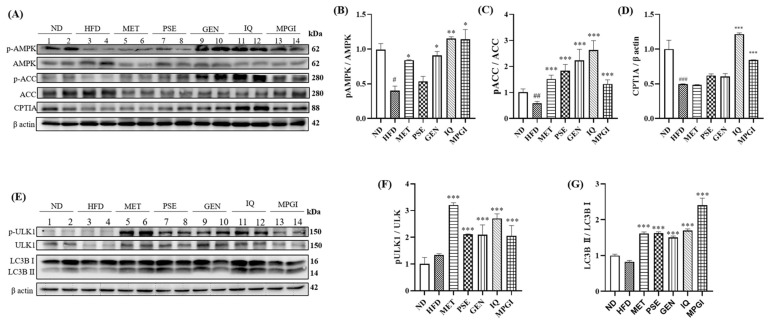
Effect of MPGI on the AMPK/ACC/CPT and AMPK/ULK1/LC3B signaling pathways in the liver. The results of Western blot analysis of p-AMPK, AMPK, p-ACC, ACC, and CPT1A in mice livers (**A**). Digitalized results of the Western blot analysis (**B**–**D**). The Western blot analysis of p-ULK1, ULK1, and LC3B in mice livers (**E**). Digitalized results of Western blot analysis in E (**F**,**G**). ND group, 1–2 wells; HFD group, 3–4 wells; MET group, 5–6 wells; PSE group, 7–8 wells; GEN group, 9–10 wells; IQ group, 11–12 wells; MPGI group, 13–14 wells. Each sample is a mixture of three independent mouse livers for each group. The data are presented as mean ± SEM. ^#^, ^##^, and ^###^ indicate significant difference at *p* < 0.05, *p* < 0.01, and *p* < 0.001 compared with the ND group; *, ** and *** indicate significant difference at *p* < 0.05, p < 0.01, and *p* < 0.001 compared with the HFD group.

As an important kinase for regulating energy homeostasis, AMPK can regulate multiple signaling pathways, such as fatty acid synthesis and autophagy, at the same time [24]. In previous studies, GEN and IQ have been determined as AMPK activators that induce autophagy and activate the AMPK signaling pathway, respectively [20,21]. Moreover, autophagy and energy metabolism play important roles in MASLD [2]. Therefore, changes in the protein levels and phosphorylation protein levels of specific proteins, such as AMPK, ACC, CPT1, ULK, and LC3B-II, located in these two signaling pathways were recorded. In the energy metabolism pathway, the phosphorylated AMPK and phosphorylated ACC in the HFD group significantly decreased (Figure 8A–C and Figure S3; *p* < 0.05 and *p* < 0.01, respectively). These two phosphorylation proteins obviously increased after the administration of MPGI or a signal molecule. Amongst them, the phosphorylation of AMPK and ACC in the GEN and IQ groups increased the most (Figure 8A,B and Figure S3A: *p* < 0.05, *p* < 0.05, *p* < 0.01, and *p* < 0.05, respectively; Figure 8A,C and Figure S3A: *p* < 0.001, *p* < 0.001, *p* < 0.001, *p* < 0.001, and *p* < 0.001, respectively), indicating that the effect of combination therapy on improving energy metabolism mainly originated from GEN and IQ. Moreover, the changes in the CPT1A protein, which is downstream of ACC in the liver, after treatment with MPGI were determined. A reduction and an increase in CPT1A protein were observed in the HFD, IQ, and MPGI groups (Figure 8A,D and Figure S3A; *p* < 0.001, *p* < 0.001, and *p* < 0.001, respectively). In the autophagy pathway, no significant difference was observed at the protein levels of phosphorylated ULK1 and LC3B II between the HFD group and the ND group. However, these phosphorylation proteins significantly increased after treatment. In particular, the effect of IQ was the strongest, reaching the same ULK1 and LC3B II phosphorylation protein levels as MET, indicating that the effect of combination therapy in increasing the autophagy level was mainly derived from IQ (Figure 8E,F and Figure S3B: *p* < 0.001, *p* < 0.001, *p* < 0.001, *p* < 0.001, and *p* < 0.001, respectively; Figure 8E,G and Figure S3B: *p* < 0.001, *p* < 0.001, *p* < 0.001, *p* < 0.001, and *p* < 0.001, respectively). These results clarified that MPGI was involved in the regulation of the AMPK/ACC/CPT1 and AMPK/ULK1/LC3B signaling pathways. 

## 3. Discussion

MASLD, which is characterized by hepatic steatosis, inflammation, and liver damage, has become a leading cause of liver transplantation and liver-associated death [2,10]. This study found that obese mice fed with a HFD diet rapidly developed severe obesity and MASLD. Moreover, MPGI was able to ameliorate hepatic steatosis and inflammation by modifying gut microbiota homeostasis, metabolites, and the TLR4/NF-κB and AMPK signaling pathways.

Currently, pharmaceutical treatment is essential in treating MASLD. However, no drug has been approved by the FDA for the treatment of this disease, and single-drug therapy for MASLD is not clinically satisfactory. Thus, the combination therapy of multiple drugs for MASLD became a considerably important research direction. PSE, GEN, and IQ come from three kinds of medicinal and food homologous materials: namely, peanut skin, *Gardenia,* and *Apocynum venetum* L., respectively. Previous studies have found that anti-obesity, antiaging and anti-oxidative stresses, and other effects can be produced via the regulation of oxidative stress, autophagy induction, and different signaling pathways, respectively [18,19,20,21]. In the present study, the different beneficial effects and action mechanisms of PSE, GEN, and IQ were utilized to combine with the optimal dosage and obtain the optimum formula for treating MASLD. Firstly, PSE, GEN, and IQ were mixed at a mass ratio of 16:10:1 to explore whether they have better antiaging activity than PSE, GEN, or IQ alone. The results in Figure 1 suggest that MPGI exhibits a better antiaging effect than a signal molecule or PSE. Secondly, a mouse model of MASLD was constructed using a high-fat feed to investigate whether MPGI could improve hepatic steatosis in mice. After 12 weeks of administrating MPGI, the changes in the body, liver, and fat weights of mice; the biochemical indices of TG, TC, ALT, and AST; and the liver histopathology (Figure 2) indicated that MPGI has a good preventive effect on MASLD in obese mice.

Other studies have recently reported that the gut–liver axis plays a vital role in liver functions, making the gut microbiota an emerging pharmacological target for liver disease [13,14]. In the present study, 16S rRNA gene sequencing was used to elucidate the effect of MPGI on gut microbiota. The results of α diversity analysis, PCoA, and an assessment of the abundance changes in the gut microbiota of the MASLD mouse model and MET and MPGI groups (Figure 3A–G) indicated that HFD induced gut microbiota dysbiosis, whereas MET and MPGI reversed it. Furthermore, the function and bacterial phenotype of the gut microbiota were predicted. Figure 4 and Figure 5 showed that the dysfunctions of Zn-related peptidase; biodegradation; transport; transcription and translation; replication; and repair and metabolism, including nucleotide, vitamin B6, and branched-chain amino acid (BCAA), are the main factors affecting the gut microbiota dysbiosis. Meanwhile, imbalances in bacterial ratio, such as increases in aerobic, Gram_negative, and biofilm_forming bacteria and reductions in anaerobic, Gram_positive, and facultatively_ anaerobic bacteria, were involved in the formation of diseases. These disorders were able to recover to normal level after administrating MPGI. 

LPS is the main metabolite of Gram-negative bacteria, and it directly binds with the TLR4 protein to stimulate the production of proinflammatory cytokines, such as TNF-α and IL-6 [23]. Inflammation is a leading feature of the progression of MASLD to MASH, and the leakage of metabolites, such as LPS, which damages the intestinal barrier, can lead to inflammation [16]. This study focused on the metabolite LPS and pro-inflammatory cytokines in the serum and liver to understand whether and how MPGI can attenuate inflammation. The changes in LPS, TNF-α, and IL-6 in serum and liver shown in Figure 6, the correlation between pro-inflammatory cytokines and gut microbiota, and the changes in the TLR4 signaling pathway shown in Figure 7 demonstrate that MPGI prevents MASLD in obese mice by inhibiting inflammation through the regulation of gut microbiota dysfunction to reduce LPS production and the modification of the TLR4/NF-κB signaling pathway.

The liver is an important organ that takes part in carbohydrate, lipid, and amino acid metabolisms [25]. The dysfunction of these metabolisms is an essential factor affecting MASLD [2,25]. AMPK is a highly conserved enzyme that regulates cellular energy homeostasis, principally by promoting glucose and fatty acid uptake [24]. Previously, GEN and IQ were found to activate AMPK [20,21]. Therefore, the present study focused on the AMPK signaling pathway to indicate whether this pathway is involved in the therapeutic effect of MPGI. The results in Figure 8 indicate that MPGI reduces hepatic lipid accumulation by activating the AMPK/ACC/CPT1A and AMPK/ULK/LC3 signaling pathways. The results are consistent with those of previous reports [20,21]. In addition, some evidence has reported that the imbalance of BCAA metabolism is closely related with MASLD [26]. This phenomenon was also observed in the present study; that is, MPGI significantly inhibited the degradation of BCAA, such as valine, leucine, and isoleucine. More interestingly, extract of milk thistle has been shown to provide liver protection in mice by promoting vitamin B12 synthesis in the gut microbiota [27]. However, MPGI inhibited the degradation of vitamin B6 to prevent HFD damage in the liver. These findings suggested that different natural products can exert hepatic protection via their different points of application, such as the synthesis and degradation of vitamin B clusters.

In normal cases, the *Akkermansia* of *Verrucomicrobia* is well-known as a beneficial bacteria, and it has been shown to significantly decrease in MASLD models of rodents [28]. However, excessive *Akkermansia* increase can lead to the excessive consumption of mucin, a reduction in microbial diversity, and the destruction of the mucus barrier, resulting in damage to the intestinal barrier, thereby inducing enteritis [28,29]. In the present study, increased abundance of *Verrucomicrobia* at the phylum level and *Akkermansia* at the genus level was observed in the HFD group. In addition, some evidence reported that *Bacillaes*, *Lactobacillaceae*, *Prevotellaceae*, *Pasterrellaceae,* and *Carnobacteriaceae* are related with the metabolism of BCAA [30]. Moreover, *Bacteroides*, *Prevotella*, *Bifidobacterium*, *Corinthia,* and *Helicobacter* may participate in the synthesis and metabolism of B6 [31]. In the present study, MPGI was shown to be involved in the metabolism of vitamin B6 via increases in *Prevotella* and *Bifidobacterium* (Supplementary Figure S4), a reduction in *Parabacteroides,* and increases in *Lactobacillaceae* and *Prevotellaceae* (Supplementary Figure S4) to inhibit the degradation of BCAA. Moreover, MPGI increased the presence of beneficial gut microbiota, such as *Lactobacillus*, *Bifidobacterium*, *Alistipes*, *Coprococcus*, *Eubacterium,* and *Faecalibacterium*, which inhibited inflammation, produced short-chain fatty acids [15,32], and reduced the presence of non-beneficial microflora, such as *Bacteroides,* to improve hepatic steatosis. These results and evidence strongly supported the conclusion that MPGI promotes beneficial gut microbes and mitigates non-beneficial ones to improve the symptoms of obese mice with MASLD via the modification of the gut–liver axis.

Taken together, the results show that combined administration is superior to single-administration therapy. MPGI effectively improved hepatic lipid accumulation in HFD-fed mice by modifying the gut microbiota; metabolites; and the TLR4/NF-κB, AMPK/ACC/CPT1A, and AMPK/ULK1/LC3B signaling pathways. In the near future, in-depth research will be performed, and whether this mixture, as a supplement, could have a therapeutic effect on patients with MASLD will be determined.

## 4. Materials and Methods

### 4.1. Drugs and Reagents

Dried peanut skin (Anjie, Fuzhou, China) was extracted using a mixture of ethanol and water at ratio of 40:60 three times. The extracts were collected and adsorbed with HP-20 resin. After the extracts were washed with water, the HP-20 resin was eluted with ethanol solution. The eluents were collected, combined, concentrated, and dried to prepare PSE. GEN, IQ, etoposide (Eto), and rapamycin were purchased from Chengdu HerbSubstance Co., Ltd., Chengdu, China. D-(+)-galactose (Sangon Biotech, Shanghai, China), D-(+)-glucose, agar (Sigma–Aldrich Co., St. Louis, MO, USA), hipolypepton (Nihon Pharmaceutical Co., Ltd., Tokyo, Japan), and yeast extract (Oxoid Ltd., Basingstoke, Hants, UK) were used to make the medium in the yeast experiment. Resveratrol was purchased from J&K Scientific Ltd., Beijing, China. A senescence-associated β-galactosidase (SA-β-Gal) kit (Beyotime Bio., Nantong, China), Dulbecco’s modified Eagle’s medium (DMEM), fetal bovine serum (CellMax Ltd., Beijing, China), horse serum, and penicillin/streptomycin (Beijing Solarbio Science & Technology Co., Ltd., Beijing, China) were used in the PC12 cell experiment. Metformin (MET) was purchased from Macklin Biochemical Co., Ltd., Shanghai, China. The ELISA kits of tumor necrosis factor α (TNF-α) and interleukin-6 (IL-6) were purchased from ExCell Bio., Ltd., Suzhou, China. The ELISA kit for LPS was provided by Hefei Laier Biotech, Anhui, China. The antibodies of Toll-like receptor 4 (TLR4), phospho-inhibitor kappa B kinase β (p-IKK β), IKK β, phospho-inhibitor kappa B α (p-IκB α), IκB α, phospho-unc-51-like kinase 1 (p-ULK1), ULK1, phospho-AMP-activated protein kinase (p-AMPK), and AMPK were purchased from Cell Signaling Technology, Danvers, MA, USA. LC3B, phospho-acetyl CoA carboxylase (p-ACC), ACC, and carnitine palmitoyltransferase1 A (CPT1A) were provided by Abcam Cambridge Biomedical Campus, Cambridge, UK. β actin and secondary antibodies (horseradish peroxidase (HRP)-linked anti-rabbit and anti-mouse IgGs) were used in this study.

### 4.2. Composition Analysis of the PSE

According to our previous results [18], the PSE mainly contains oligosaccharides and non-oligosaccharides. After careful analysis of the ^1^H and ^13^C NMR spectra of the oligosaccharides part of the PSE [18], we propose that the main ingredient of the PSE oligosaccharides is sucrose. To confirm this result, the oligosaccharides part was acetylated (1 mL pyridine: 1 mL acetic anhydride) to afford the derivatives. Then, the acetylated derivatives of oligosaccharides were purified via HPLC (Supersil Phenyl 5 μm (ϕ 10 × 200 mm), Elite, flow rate: 3 mL/min, detected at 210 nm, 40%–100% aqueous MeOH (containing 0.1% formic acid), 30min, linear gradient elution) to obtain a pure acetylated PSE oligosaccharide derivative (*t*_R_ = 22–24 min). The ^1^H NMR spectrum and MS data of the PSE oligosaccharide acetylated derivative were identical to those of the ^1^H NMR spectrum and the MS data of acetylated authentic sucrose (Supplementary Figure S5). HRESI-TOF-MS (M + Na) ^+^ *m*/*z* 701.1952, which were calculated for C_28_H_38_NaO_19_ (M + Na) ^+^ 701.1900. In order to determine the chemical structure of the oligosaccharides part of PSE more accurately and purify the non-oligosaccharides part, PSE was again purified according to HPLC conditions in our published results. The **Compound 1** (*t*_R_ = 5–6 min) was obtained from the oligosaccharides PSE part. The ^1^H NMR spectrum of **Compound 1** was identical to that of authentic sucrose (Supplementary Figure S6). The **Compound 2** (*t*_R_ = 20–21 min) and **Compound 3** (*t*_R_ = 58–59 min) were obtained from the non-oligosaccharides part of the PSE, and the ^1^H NMR and MS spectra were measured (Supplementary Figures S7 and S8). **Compound 2** was identified as (+)-catechin by comparing the ^1^HNMR data with those reported in literature [33]. ^1^H NMR (500 MHz, Acetone-*d*_6_) *δ* 6.90 (d, *J* = 2.0 Hz, 1H), 6.80 (d, *J* = 8.1 Hz, 1H), 6.76 (dd, *J* = 8.1, 2.0 Hz, 1H), 6.03 (d, *J* = 2.3 Hz, 1H), 5.89 (d, *J* = 2.3 Hz, 1H), 4.56 (d, *J* = 7.8 Hz, 1H), 3.99 (m, 1H), 2.92 (dd, *J* = 16.1, 5.6 Hz, 1H), 2.53 (dd, *J* = 16.1, 8.4 Hz, 1H). HRESI-TOF-MS (M + H) ^+^ *m*/*z* 291.0834, which was calculated for C_15_H_15_O_6_ (M + H) ^+^ 291.0863. **Compound 3** was identified as proanthocyanidin A-1 by comparing the ^1^H NMR data with those reported in literature [34]. ^1^H NMR (400 MHz, Methanol-*d*_4_) *δ* 7.13 (d, *J* = 1.6 Hz, 1H), 7.01 (dd, *J* = 8.3, 1.6 Hz, 1H), 6.91 (s, 1H), 6.80 (d, *J* = 7.1 Hz, 3H), 6.08 (s, 1H), 6.06 (d, *J* = 2.0 Hz, 1H), 5.95 (d, *J* = 2.0 Hz, 1H), 4.72 (d, *J* = 7.8 Hz, 1H), 4.23 (d, *J* = 3.3 Hz, 1H), 4.14 (m, 1H), 4.06 (d, *J* = 3.3 Hz, 1H), 2.94 (dd, *J* = 16.4, 5.5 Hz, 1H), 2.57 (dd, *J* = 16.4, 8.3 Hz, 1H). HRESI-TOF-MS (M + H) ^+^ *m*/*z* 577.1345, which was calculated for C_30_H_25_O_12_ (M + H) ^+^ 577.1341.

### 4.3. Replicative Lifespan Assay of K6001 Yeast Strain

The K6001 yeast biosystem was used in this experiment to evaluate the antiaging activity of the components. Firstly, K6001 yeast cells stored at −30 °C were inoculated into 5 mL liquid galactose medium and incubated at 28 °C with shaking for 24 h. The next day, 5 mL glucose medium was poured into glass dishes, and after the medium solidified, 150 μL ethanol or different concentrations of the sample solutions were added to each dish. After the solvent evaporated, 1 mL of yeast cells cultured for 24 h was placed into a centrifuge tube and washed three times with PBS. The washed yeast cells were counted using a hemocytometer, and approximately 4000 yeast cells were evenly spread on each dish and incubated at 28 °C for 48 h. Forty microcolonies were randomly selected from each dish under a microscope, and the number of daughter cells in each colony was calculated. In this experiment, a 2.28 μg/mL concentration of resveratrol was used as a positive control.

### 4.4. SA β-Gal Assay of PC12 Cells

PC12 cells were purchased from the cell bank of the Chinese Academy of Sciences, Shanghai, China. Approximately 50,000 of the PC12 cells were seeded in 24-well plates containing 1 mL CM (DMEM containing 10% horse serum, 7.5% fetal bovine serum, and 1% Penicillin/Streptomycin). After the cells were incubated at 37 °C for 24 h in a CO_2_ incubator, the CM was replaced with EM (DMEM containing 1% Penicillin/Streptomycin) containing different concentrations of the sample. Subsequently, the cells were incubated for 1 day in a 37 °C incubator, the culture medium was replaced with EM containing 0.558 µg/mL Eto, and the cells were incubated for a further day (the control group was replaced with EM). The culture solution was aspirated, and 200 μL of 4% paraformaldehyde was added to each well after PBS washing. The cells were fixed at room temperature for 15 min, and the cell fixative solution was aspirated. Then, 200 μL staining working solution, which contained 10 μL β-Gal staining solution A, 10 μL β-Gal staining solution B, 930 μL β-Gal staining solution C, and 50 μL X-Gal solution, was added to each well after PBS washing had been conducted three times. The 96-well plate was sealed with plastic wrap to avoid the solution evaporating and incubated in a CO2-free incubator overnight at 37 °C. Afterwards, the cells were observed under microscope. The proportion of cells stained blue in the field of view was calculated as a proportion of all cells. In this experiment, rapamycin was used as the positive control.

### 4.5. Animal and Experimental Design

MET, PSE, GEN, IQ, and MPGI were dissolved in water containing 1% DMSO prior to oral administration. A total of 70 6-week-old Institute of Cancer Research (ICR) mice were purchased from Zhejiang Academy of Medical Sciences, Hangzhou, China. They were acclimatized in a standard condition, at a temperature of 23 °C, with a humidity of 55%, and with a 12 h light/dark cycle. After 1 week of acclimatization, the mice were randomly divided into seven groups: normal diet (ND) group, high-fat diet (HFD) group, HFD + 140 mg/kg MET group, HFD + 80 mg/kg PSE group, HFD + 50 mg/kg GEN group, HFD + 5 mg/kg IQ group, and HFD + 80 mg/kg PSE + 50 mg/kg GEN + 5 mg/kg IQ (MPGI) group. The dose used in each group was the optimal dose determined in previous studies [18,19,20,21]. The ND group was given a chow diet and vehicle (water containing 1% DMSO), whereas the others were given HFD food (TP23300, Trophic Animal Feed High-tech Co., Ltd., Nantong, China) and the respective drug dosage via oral gavage daily for 12 weeks. All the animal experiments were performed in accordance with the guidelines of the National Institutes of Health for the care and use of laboratory animals. Standard experimental protocols were used in accordance with the requirements of the Ethics Committee for animal experiments of Zhejiang University (Permit number: ZJU20220334). At the end of the experiment, the plasma and tissues, such as those of heart, liver, spleen, kidney, intestine, and brain, were obtained, weighed, and stored in a −30 °C freezer for further analysis. The plasma samples of mice were placed for 0.5 h and then centrifuged at 12,000 rpm at 4 °C to obtain serum. 

### 4.6. Biochemical Analysis and Immunological Staining

Approximately 200 µL of serum was collected to measure triglyceride (TG), total cholesterol (TC), alanine amino transferase (ALT), and aspartate amino transferase (AST) using an automatic biochemical analyzer. Eight mice from each group were used for the measurements, which were carried out by HaoKe Bio. Co. Ltd., Hangzhou, Zhejiang, China. Additionally, three mouse liver samples were randomly selected from each group for histological staining. For hematoxylin and eosin (H&E) staining, the liver samples were fixed in 4% paraformaldehyde for 48 h, dehydrated with an alcohol gradient, and were then made transparent with xylene. Afterwards, they were embedded with paraffin, cut into slices with a thickness of 5 µm using a paraffin slicing machine (Leica RM2235, Leica Biosystems Ltd., Beijing, China), and stained with H&E. For oil red O staining, the liver samples were dehydrated with a gradient of 15% and 30% sucrose solution, embedded with OCT, cut into frozen sections with a thickness of 10 µm, and then stained with oil red O. Afterwards, they were observed under the microscope. 

### 4.7. Enzyme-Linked Immunosorbent Assay (ELISA)

Serum, liver, and fecal samples were collected from the mice at the end of the experiment and stored at −80 °C for biochemical analysis. Samples from six mice in each group were used for detection. For the liver samples, 15–30 mg of mouse liver tissue was added to 500 μL of PBS and 5 μL of protease inhibitor, homogenized, and centrifuged at 12,000 rpm for 4 °C to collect the supernatant as protein sample. Subsequently, the concentrations of the protein samples were measured with a BCA assay kit. Fecal samples were prepared as described in a previous study [35]. Briefly, mouse feces were collected at 12 weeks after administering PSE, GEN, IQ, and MPGI during the animal experiment. Then, 30–60 mg fecal samples from each group were added to 1 mL of PBS, homogenized, and centrifuged. The supernatants of the feces were filtered using a 0.22 μm filter membrane and inactivated in a 90 °C water bath for 15 min. The supernatants of the control and treatment groups were diluted with PBS at ratios of 1:5 and 1:10, respectively.

The LPS, TNF-α, and IL-6 concentrations of the samples were measured using commercially available mouse ELISA kits in accordance with the manufacturer’s instructions. The sample and standard were added to the respective wells, followed by the addition of biotin conjugate. They were mixed properly and incubated in a 300 rpm shaker at room temperature for 2 h. Afterwards, the wells were washed with washing buffer five times and -streptavidin conjugate was added. The wells were incubated in a 300 rpm shaker at room temperature for 1 h and then washed five times. After the substrate solution was added and incubated at 37 °C for 15 min, the stop solution was added and the optical density was detected using a microplate reader (BioTek, San Diego, CA, USA) at 450 nm. The concentrations of LPS, TNF-α, and IL-6 were calculated based on their respective standard curve.

### 4.8. Western Blot Analysis

Three liver samples from each group of mice were randomly selected and homogenized in RAPI buffer (Beijing ComWin Biotech Co., Ltd., Beijing, China) containing 1% protease inhibitor (Beijing ComWin Biotech Co., Ltd., Beijing, China) and 1% phosphatase inhibitor (Abcam Cambridge Biomedical Campus, Cambridge, UK), followed by determination of protein concentration. Subsequently, approximately 20 µg of protein from each sample was mixed with 5× SDS-PAGE loading buffer and heated at 100 °C for 10 min prior to loading onto sodium dodecyl sulphate–polyacrylamide gel. Then, gel electrophoresis was conducted at 80 V for 15 min and 120 V for 60 min. Subsequently, the protein was transferred from the gel to a polyvinylidene difluoride membrane, which was subsequently blocked with 5% non-fat dry milk buffer for 80 min at room temperature. The primary antibody (TLR4, p-IKK-β, IKK-β, p-IκB -α, IκB-α, p-ULK1, ULK1, p-AMPK, AMPK, LC3B, p-ACC, or ACC) was added to the universal antibody diluent (New Cell & Molecular Bio. Co. Ltd., Suzhou, China) at a ratio of 1:1000 and incubated with membrane overnight at 4 °C. After the membrane was washed three times with TBST, the secondary antibody (HRP-linked anti-mouse or anti-rabbit IgGs) was applied for 45 min. The protein bands were detected using an ECL Western blot chemiluminescence detection kit (Vazyme Bio. Co., Ltd., Nanjing, China), and the blot density was analyzed using Image Lab software version 6.1.0 (Bio-Rad, Hercules, CA, USA).

### 4.9. Gut Microbiota Analysis

Fresh fecal samples from each mouse collected during week 8 of the animal experiment were used for gut microbiota analysis. These samples, from eight mice in the ND, HFD, MET, PSE, and MPGI groups, were randomly selected for analysis. Firstly, the total genomic DNA was extracted using the TIANamp Bacterial DNA kit, followed by amplification of the V4 regions of the 16S rRNA gene by using composite sense and anti-sense primers under appropriate PCR conditions. Afterwards, the broken sticky end of the target amplicon fragment was repaired using Klenow DNA polymerase, T4 DNA polymerase, and T4 PNK. Magnetic beads were then utilized to purify the amplicons, which were pooled after replication. Subsequently, the sequences were analyzed using the DAD2 method (Benjamin J Callahan, Stanford University, USA), and amplicon sequence variants (ASVs) were assigned to each representative sequence. The analysis was carried out by LC-Bio Technology Co., Ltd., Hangzhou, China.

### 4.10. Biostatistical Analysis

Data were analyzed using one-way or two-way ANOVA followed by a Dunnet t-test on GraphPad Prism software (version 8.0; GraphPad Prism, San Diego, CA, USA). Statistical analysis on gut microbiota was conducted using R software (version 4.2.3). All data are expressed as mean ± SEM, and differences at *p* < 0.05 were considered statistically significant.

## Data Availability

The data presented in this study are available upon request from the corresponding author.

## Supplementary Materials

The following supporting information can be downloaded at: https://www.mdpi.com/article/10.3390/ijms242316684/s1.

## Abbreviations

alanine amino transferase, ALT; amplicon sequence variants, ASVs; aspartate amino transferase, AST; branched-chain amino acid, BCAA; carnitine palmitoyltransferase1 A, CPT1A; Dulbeccor’s modified Eagler’s medium, DMEM; enzyme-linked immunosorbent assay, ELISA; etoposide, Eto; geniposide, GEN; hepatocellular carcinoma, HCC; hematoxylin and eosin, H&E; high-fat diet, HFD; interleukin 6, IL-6; isoquercitrin, IQ; microtubule-associated protein 1A/1B-light chain 3B, LC3B; lipopolysaccharide, LPS; metabolic-dysfunction-associated steatohepatitis, MASH; metabolic-dysfunction-associated steatotic liver disease, MASLD; metformin, MET; mixture of peanut skin extract, geniposide, and isoquercitrin, MPGI; non-alcoholic fatty liver disease, NAFLD; normal diet, ND; phospho-acetyl CoA carboxylase, pACC; phospho-AMP-activated protein kinase, pAMPK; peanut skin extract, PSE; phospho-inhibitor kappa B-α, pIκB-α; phospho-inhibitor kappa B kinase β, pIKK β; phospho-unc-51-like kinase 1, pULK1; senescence-associated β-galactosidase, SA β- Gal; short-chain fatty acid, SCFA; total cholesterol, TC; triglycerides, TG; toll-like receptor 4, TLR4; tumor necrosis factor alpha, TNF-α.
