# Supplementary material for: Mixture of Peanut Skin Extract, Geniposide, and Isoquercitrin Improves the Hepatic Lipid Accumulation of Mice via Modification of Gut Microbiota Homeostasis and the TLR4 and AMPK Signaling Pathways
Source: Int J Mol Sci. 2023 Nov 24;24(23):16684. doi: 10.3390/ijms242316684 (PMC10706562; doi:10.3390/ijms242316684)
Supplement: Supplementary file 1 [file ijms-24-16684-s001.zip › ijms-2698769-supplementary.pdf]

Supplementary information

Article

Mixture of Peanut Skin Extract, Geniposide, and Isoquercitrin Improves the Hepatic Lipid Accumulation of Mice via Modification of Gut Microbiota Homeostasis and the TLR4 and AMPK Signaling Pathways

Meijuan Yi [1], Opeyemi B. Fasina [1], Yajing Li [1], Lan Xiang [1,*] and Jianhua Qi [1,2,*]

[1] College of Pharmaceutical Science, Zhejiang University, Hangzhou 310058, China; 22119153@zju.edu.cn (M.Y.); fasinaopeyemi@zju.edu.cn (O.B.F.); 12019045@zju.edu.cn (Y.L.)
[2] Jinhua Institute of Zhejiang University, Jinhua 321299, China
* Correspondence: lxiang@zju.edu.cn (L.X.); qijianhua@zju.edu.cn (J.Q.)

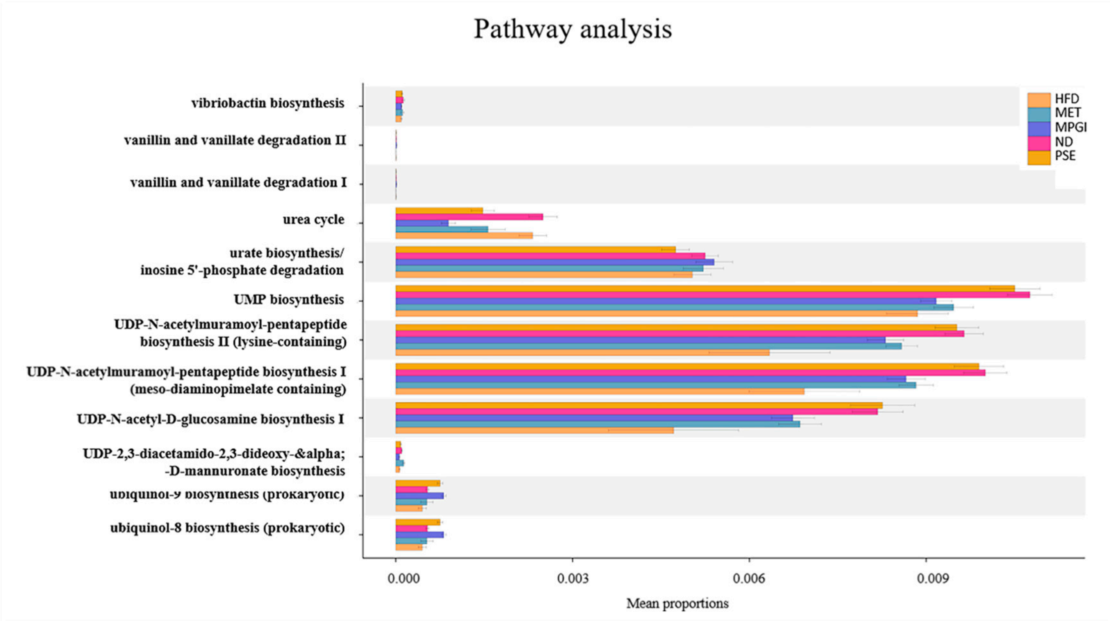

Supplementary Figure S1. Pathway analysis of function prediction for the gut microbiota after administrating MPGI with PICRUSt2 software.

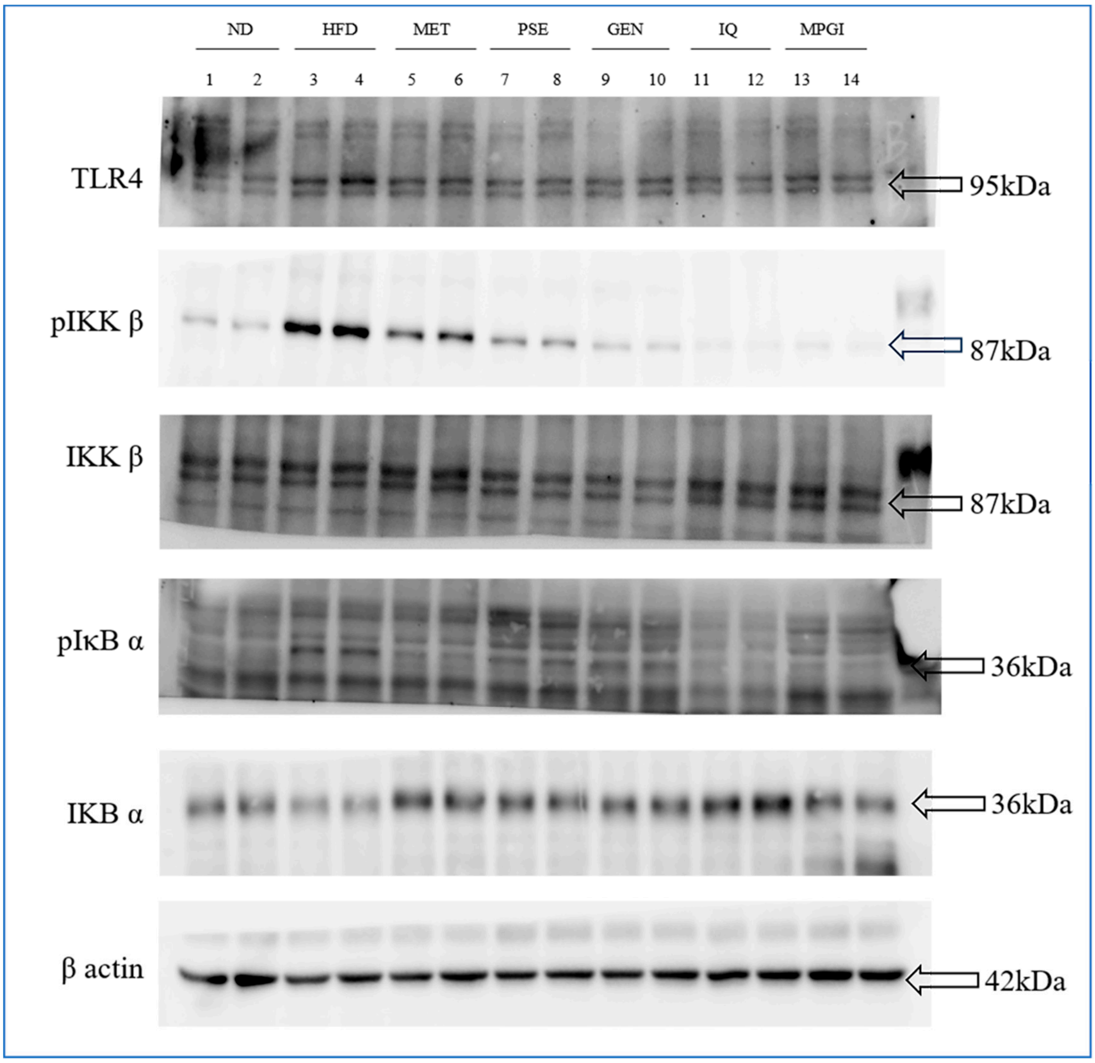

Supplementary Figure S2. Original western blot analysis of Figure 7. Original western blot of TLR4, pIKK β, IKK β, pIκB α, IκB α and β actin in liver of mice in Figure 7.

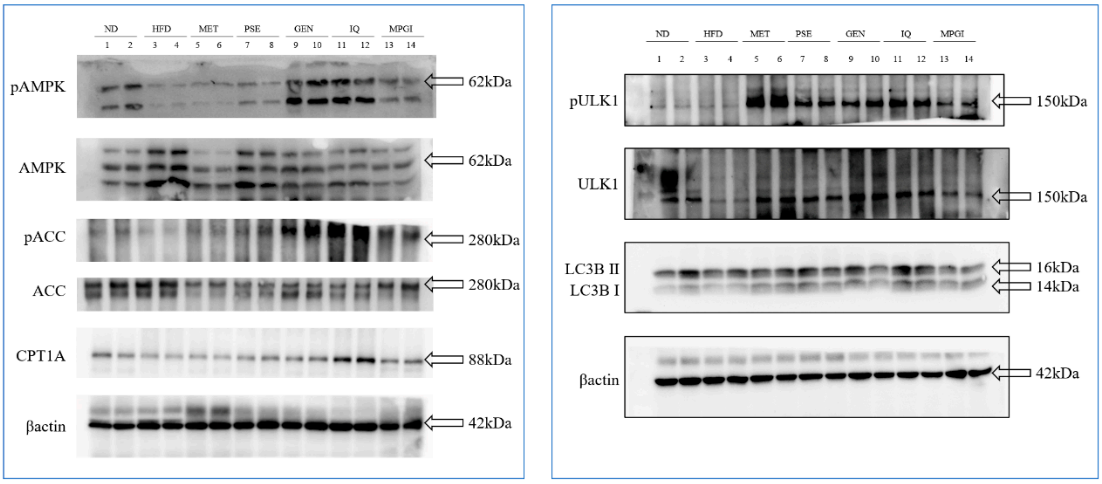

Supplementary Figure S3. Original western blot analysis of Figure 8. Original western blot of phosphate-AMPK, AMPK, phosphate-ACC, ACC, CPT1A and β-actin in Figure 8(A), and phosphate-ULK1, ULK1, LC3B and β-actin of in mice liver in Figure 8(B).

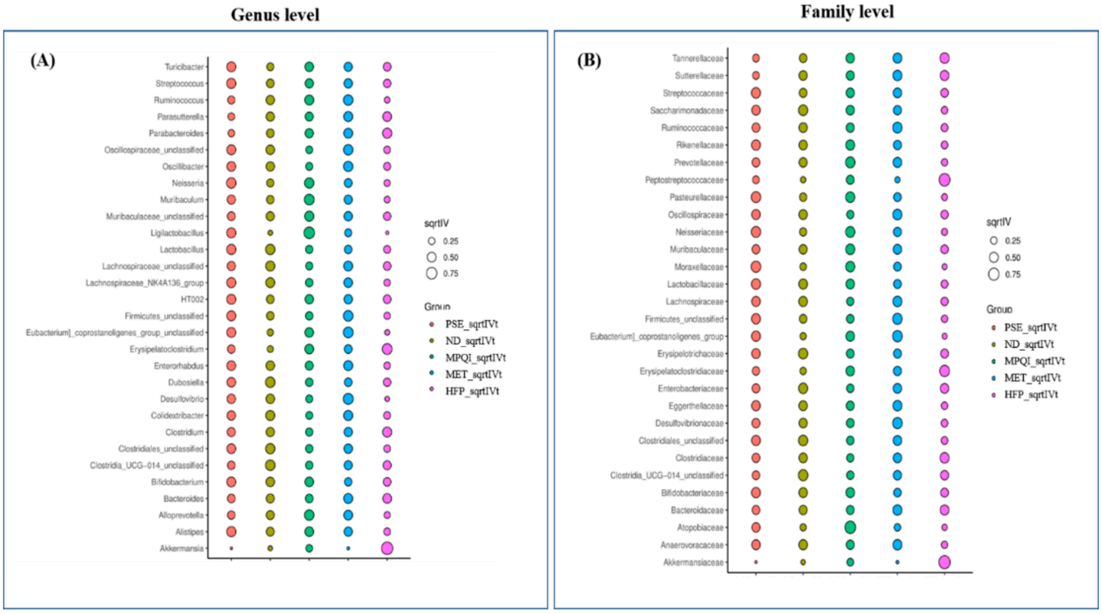

Supplementary Figure S4. Advanced analysis for biomarkers. The changes on bio-indicators of each group after administrating MPGI at genus (A) and family (B) levels.

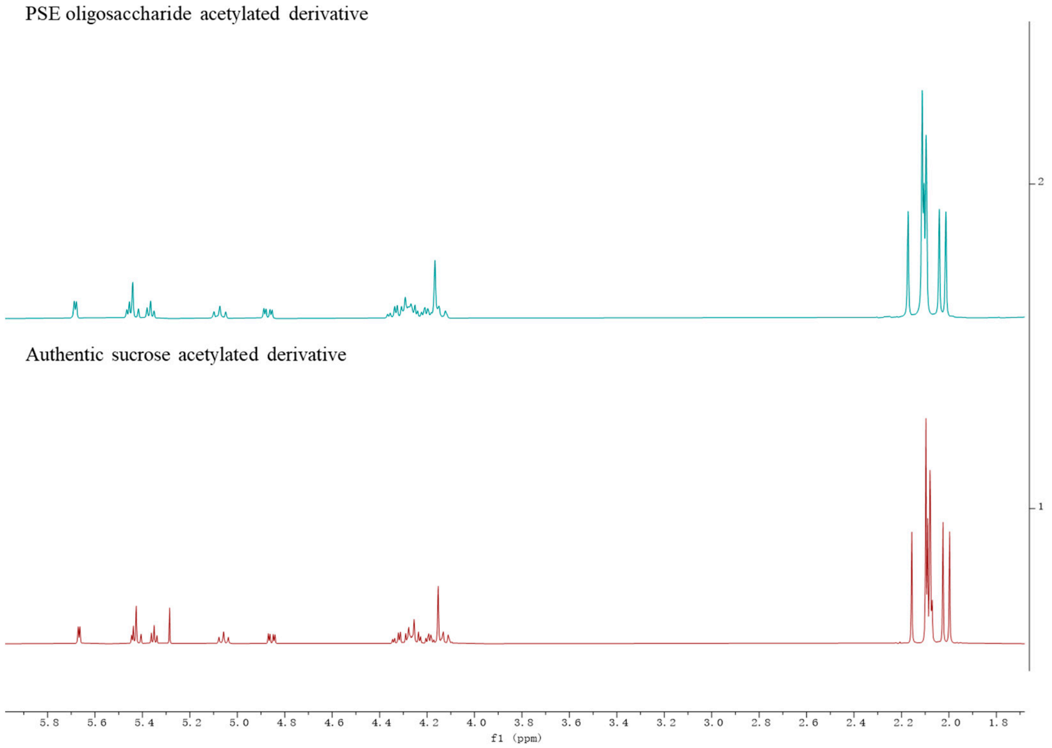

Supplementary Figure S5. Comparison of ^{1}H NMR spectra of PSE oligosaccharide acetylated derivative with acetylated derivative of authentic sucrose.

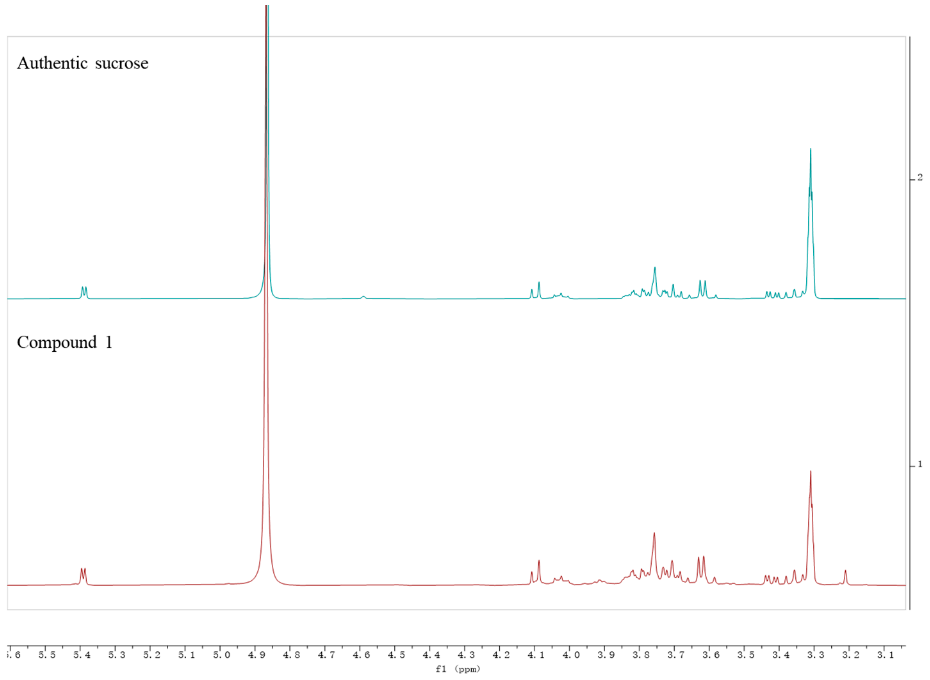

Supplementary Figure S6. Comparison of ^{1}H NMR spectra of Compound 1 with authentic sucrose.

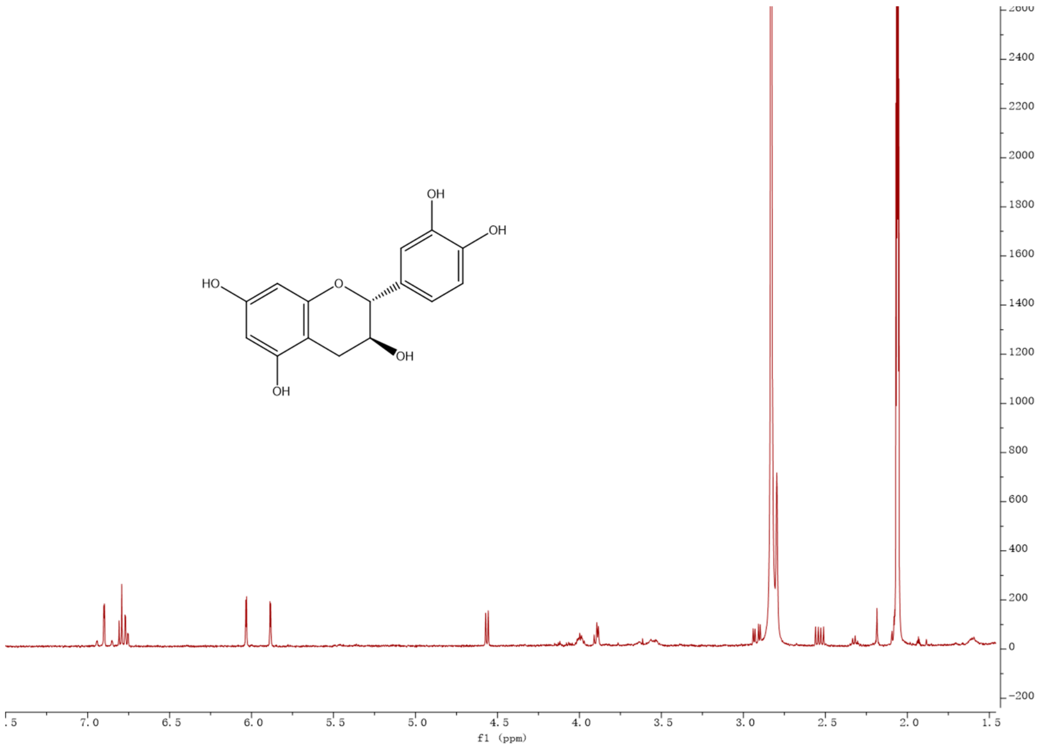

Supplementary Figure S7. The ^{1}H NMR spectrum of compound 2.

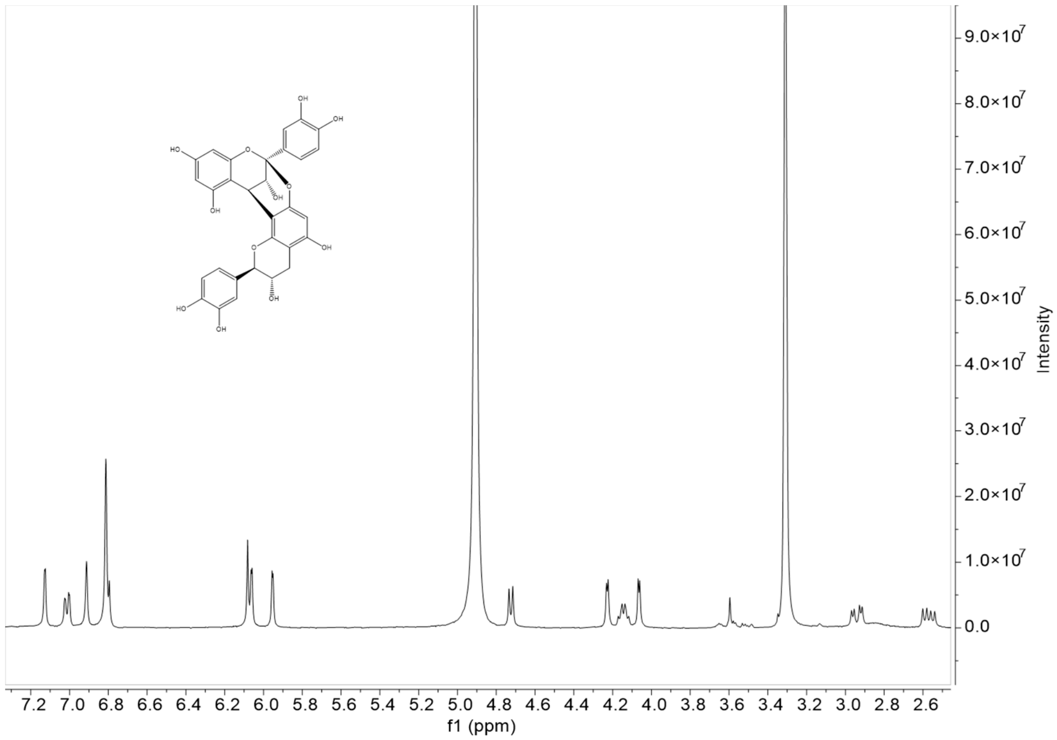

Supplementary Figure S8. The ^{1}H NMR spectrum of compound 3
